# Insufficient compliance of the German Therapeutic Products Advertising Act in product catalogues of online pharmacies

**DOI:** 10.1007/s00210-024-03370-7

**Published:** 2024-08-29

**Authors:** Lara Barlage, Roland Seifert

**Affiliations:** Institute of Pharmacology, Hannover Medical School, 30625 Hannover, Germany

**Keywords:** Online pharmacy, Drug advertising, Advertisement, Pharmacy review, Therapeutic Products Advertising Act (Drugs Advertising Law), Law review, Self-medication, Pubmed.gov

## Abstract

The importance of ordering drugs from online pharmacies in Germany is increasing constantly. At the same time, there are many online pharmacies that try to increase their own market share through advertising. In Germany, the advertising of drugs is regulated by the Therapeutic Products Advertising Act (*Heilmittelwerbegesetz* (HWG)) and must therefore also be complied with by online pharmacies in their product catalogues. One important purpose of the HWG is to protect consumers by ensuring that they are presented with all necessary medical-pharmacological information about the advertised product. This paper examines the implementation of as well as the compliance with Section 4 of the HWG in the product catalogues of two online pharmacies. For this purpose, the spring/summer catalogue of 2023 was considered in the allergy, cold and gastrointestinal tract categories, resulting in the inclusion of 143 drugs from online pharmacy 1 (OP1) and 102 drugs from online pharmacy 2 (OP2) for the analysis. The information on the drugs was taken from the respective catalogue and the pharmaceutical index ‘Gelbe Liste’, collected in tabular form and was encoded. Subsequently, the required mandatory information according to Section 4 was checked by comparing the collected information with the information from the package leaflet. The analysis revealed that both catalogues insufficiently complied with Section 4 of the HWG. OP1 complied with 75% of the mandatory information required under Section 4 of the HWG and OP2 with 64%. In the OP1 catalogue, there is no indication for 6% of the drugs and no specification for 29% of the traditionally registered drugs. In addition, the mandatory notice on risks and adverse effects in the OP1 catalogue is not presented in a consumer-friendly way. In the OP2 catalogue, further concerns about the compliance with the HWG were raised due to the missing indication for 17% of the drugs, no warning for 64% of the ethanol-containing drugs and no active ingredient for 31% of the monopreparations. The insufficient compliance with the HWG in the product catalogues of OP1 and OP2 means that consumer protection as a stated objective of the HWG cannot be guaranteed. Advertising for drugs should only be possible under the condition that the content of the advertisement complies with the HWG. Insufficient compliance, as in the product catalogue of OP1 and OP2, should be prohibited as it directly contradicts the law’s objective.

## Introduction

Online pharmacies are popular globally. While the average revenue per user was 16.41 euros in 2017, this number had already risen to 25.06 euros in 2023 and is expected to reach 30.11 euros in 2028 (https://de.statista.com/outlook/hmo/digital-health/digitale-behandlung-pflege/digital-care-management/online-apotheke/weltweit#umsatz; last accessed 13 April 2024). A study by the International Pharmaceutical Federation (FIP) analysed the worldwide online pharmacy phenomenon in 2021. One hundred eighteen countries were contacted, of which 79 responded and were included in the analysis (Dineen-Griffin and Usman 2021). It was analysed whether non-prescription products can be purchased online. It was concluded that it is possible in 61% of the participating countries through brick-and-mortar pharmacies with an associated online shop and in 15% through independent online pharmacies, and that in 19%, it is not permitted at all (Dineen-Griffin and Usman 2021). Selling prescription products online is forbidden in 25% of the participating countries, whereas 34% of them allow an online sale if the online pharmacy is part of a brick-and-mortar pharmacy (Dineen-Griffin and Usman 2021). In Germany, it is permitted to offer both prescription and non-prescription products via online pharmacies. This makes Germany one of the seven member states in the EU that have not imposed a ban on the mail-order sale of prescription-only drugs (ABDA – Bundesvereinigung Deutscher Apothekerverbände e. V., 2023). The majority of countries (51%) stated that they have no laws for online pharmacies, whereas the remaining countries do have existing regulations and laws, which apply to 74% of European countries (Dineen-Griffin and Usman 2021).

In 2022, there were 3125 pharmacies in Germany with a mail-order license, of which only around 150 pharmacies actively offered mail-order sales (ABDA – Bundesvereinigung Deutscher Apothekerverbände e. V., 2023). On average, 62% of the German population regularly shopped at online pharmacies in 2021 (https://www.bitkom.org/Presse/Presseinformation/Rueckenwind-fuer-Online-Apotheken-durch-Corona; last accessed 10 June 2024). This underlines the importance of online pharmacies in Germany and shows that they have become indispensable in our digital age.

Since there is a lot of competition in the online pharmacy sector and most pharmacies generally offer similar products, advertising must be used to attract the attention of consumers (Fritz et al. 2021). All advertising for drugs must comply with the German Therapeutic Products Advertising Act (*Heilmittelwerbegesetz* (HWG)). The HWG consists of 18 sections and came into force on 15 July 1965 (Doepner 2000). Section (Sect.) 4 of the HWG only applies to drugs within the meaning of Sect. 2 of the German Drug Act (*Arzneimittelgesetz* (AMG)) and regulates the mandatory information that must be included in the advertising of drugs. This is intended to ensure that the consumer receives the necessary information about all pharmacological characteristics of the product and consequently has the opportunity to assess its suitability for his specific needs as well as the necessity of purchasing it (Die Bundesregierung der Bundesrepublik Deutschland 1975; Doepner and Reese 2023). Thus, Sect. 4 of the HWG is intended to protect the consumer from unreflected and unnecessary purchases of drugs by providing pharmacological information (Doepner and Reese 2023). The HWG states that less mandatory information is stipulated for advertising aimed at laypersons than for advertising aimed at healthcare professionals.

Studies on drug advertising in print media were performed. Sansgiry et al. (1999) analysed print drug advertising for over-the-counter (OTC) products aimed at laypersons in the USA. More than 50% of the advertisements analysed did not provide the consumer with the necessary information for assessing the product’s suitability for their own purposes (Sansgiry et al. 1999). Solhaug et al. (2006) also concluded that only about 50% of written pharmaceutical advertising to doctors in Norway was legally compliant. Although little of the information was false, the statements were presented in a favourable way (Solhaug et al. 2006).

A study by Keuper and Seifert (2023) deals with the German HWG, which analysed whether the HWG was complied with in the highly popular German consumer health magazine ‘Apotheken Umschau’. The HWG was largely disregarded in that specific magazine, regarding the provision of warnings and mandatory notices on risks as well as adverse effects. In contrast, information on indications and product names was compliant with the law (Keuper and Seifert 2023).

These studies show that there are severe deficiencies in the implementation of drug advertising across countries. There is little literature on pharmaceutical advertising for laypersons and little international literature that deals with the German HWG apart from the study by Keuper and Seifert (2023). In this study, we provide an in-depth analysis of the compliance with and the implementation of Sect. 4 of the HWG in the product catalogues of two online pharmacies in Germany.

## Material and methods

### Online pharmacies and product catalogues

We began our project by identifying online pharmacies that offer product catalogues and then decided to select the top-selling online pharmacy that offers a product catalogue which is denoted as online pharmacy 1 (OP1) in this paper. In 2023, the top 15 online pharmacies in Germany generated total net sales of 2830 million euros, of which over 19%, 550 million euros, were generated by OP1 (Sempora Consulting GmbH 2024). Additionally, we selected a smaller online pharmacy for comparison, referred to as online pharmacy 2 (OP2). OP2 generated net sales of EUR 85 million in 2021, which is 3% of the sales of the top 15 online pharmacies (Sempora Consulting GmbH 2024). This paper aims to present the implementation of the HWG in advertising catalogues of online pharmacies in a generalized context, rather than focusing on specific pharmacies. Therefore, the online pharmacies are denoted as OP1 and OP2.

This analysis draws from the Spring/Summer 2023 product catalogues of the two online pharmacies. The catalogues are available free of charge online. The OP1 catalogue was available as a PDF file for free during the validity period, whereas the OP2 catalogue was available as an online flip catalogue as well as a PDF file during the validity period. In addition, the catalogues could be ordered free of charge as an analogue catalogue from the respective online pharmacy during the period of validity. After having reviewed both catalogues, we focused our analysis to allergies, colds and gastrointestinal tract. In order to select the three categories to be analysed, we used the top 10 list of the top-selling indication groups for OTC drugs from the report by the German Association of Pharmaceutical Manufacturers (Bundesverband der Arzneimittel-Hersteller e.V.) for 2021 (Bundesverband der Arzneimittel-Hersteller e.V., 2022). We selected first place (cold remedies), fourth place (gastrointestinal tract remedies) and tenth place (antiallergics) from the list considering that these categories were sufficiently represented in both catalogues (Bundesverband der Arzneimittel-Hersteller e.V., 2022).

### HWG validity

This paper analyses the product catalogues of the two online pharmacies for compliance with the requirements of the HWG. The subsequent analysis critically examines Sect. 4 of the HWG, which delineates the requisite mandatory information. As it only refers to drugs specified in Sect. 2 of the AMG, it does not apply to other products such as medical devices, cosmetics or food supplements. Furthermore, considering that all promoted drugs are non-prescription and accessible over the counter, with the intended audience being laypersons, the analysis exclusively incorporates legal provisions applicable to these criteria. The examination proceeds systematically, presenting and scrutinizing the provisions of Sect. 4 of the HWG in chronological order. Table 1 shows the parameters analysed in this paper. The third column presents an interpretation of the legal provision in accordance with the legal commentary. These parameters represent the legal requirements that must be observed by the two online pharmacies in their product catalogues.

**Table 1:** Overview of the analysed parameters and their significance

Analysed parameters	Legal passage HWG	Explanation
Brand name of the drug	Section 4 (1) sentence 1 no. 2	The brand name of the drug must be listed and correspond to the name at the time of authorization (Doepner and Reese 2023)
Indications	Section 4 (1) sentence 1 no. 4	Indications that are advertised must be stated, and only those that were also recorded at the time of approval (Doepner and Reese 2023). In the case of homeopathy, an indication may only be stated for products that are exempt from registration or authorized; otherwise, Sect. 5 of the HWG applies (Doepner and Reese 2023)
Warnings	Section 4 (1) sentence 1 no. 7	Only warnings that are printed on the outer packaging of the product in accordance with Sect. 10 of the AMG must be included in the advertising, so that warnings from the package leaflet do not have to be included in the advertising (Prütting 2016)
Traditionally registered drugs	Section 4 (1) sentence 2	In the case of registered traditional herbal products, a reference must be made to the fact that the product is only used for specific illnesses because of many years of use (Doepner and Reese 2023). In this context, an area of application may be listed in the labelling (Sect. 4 (1) sentence 2 of the HWG)
Monopreparations	Section 4 (1a)	In the case of monopreparations, the active substance must follow the brand name of the drug in the short term, unless the brand name already contains the active substance (Doepner and Reese 2023). A clear classification as an active ingredient must be evident (Doepner and Reese 2023)
Mandatory notice	Section 4 (3)	In the case of advertising for laypersons, the statement ‘Zu Risiken und Nebenwirkungen lesen Sie die Packungsbeilage und fragen Sie Ihren Arzt oder Apotheker’ must be included (Doepner and Reese 2023). This must be listed separately from other advertising contents and must be clearly legible (Doepner and Reese 2023) But if the product is an OTC product which is not only available in pharmacies, the notice also only needs to be included if the risks and adverse effects of these products are noted on the packaging or in the package leaflet (Sect. 4 (3) sentence 4 of the HWG)
Formal requirements	Section 4 (4)	The mandatory information required under Sect. 4 (1) of the HWG must be listed separately from other advertising contents and must be clearly distinguishable from it (Sect. 4 (4) of the HWG). Good legibility must be ensured (Sect. 4 (4) of the HWG)
Reminder advertising	Section 4 (6)	Reminder advertising must not contain any medical or pharmacological content (Doepner and Reese 2023). The legal definition also permits the company name, trademark of the pharmaceutical company and active ingredient in addition to the brand name of the drug (Sect. 4 (6) sentence 2 of the HWG). Moreover, information on package size and sales price is permitted as it does not contain any pharmacological content (Doepner and Reese 2023)

### Data collection

The products advertised in the three categories were recorded in an initial overview table. Information on the product name, the pharmaceutical central number (PZN), pharmaceutical form, active ingredient, warnings, indications as well as authorized age were listed, provided the necessary information was given in the respective catalogue. It was also documented whether the mandatory notice on risks and adverse effects was given for the product. A total of 175 products from the OP1 catalogue and 117 products from the OP2 catalogue were tabulated. The intersection of the common products in the two catalogues amounted to a total of 53 products from all three categories. We decided to include all products that were offered in the respective catalogue for the three defined categories in the analysis, as the focus of this paper is not to be on the products themselves, but rather on the generalized implementation of the HWG in product catalogues.

These overview tables were supplemented with information taken from the Pharma Index ‘Gelbe Liste’ (https://www.gelbe-liste.de/; last accessed 18 December 2023) on the pharmacy obligation, the product type and the mode of action. The overview tables on the contents of the catalogues were then modified separately for each catalogue by removing products other than drugs specified in Sect. 2 of the AMG from the tables so that only drugs were listed. As a result, the analysis still included 143 drugs from OP1 and 102 drugs from OP2. These numbers represent the basic quantity from which comparisons and calculations were made, unless otherwise stated. As the basic quantities have different sizes, relative numbers were used for better comparison.

**Fig. 1:**
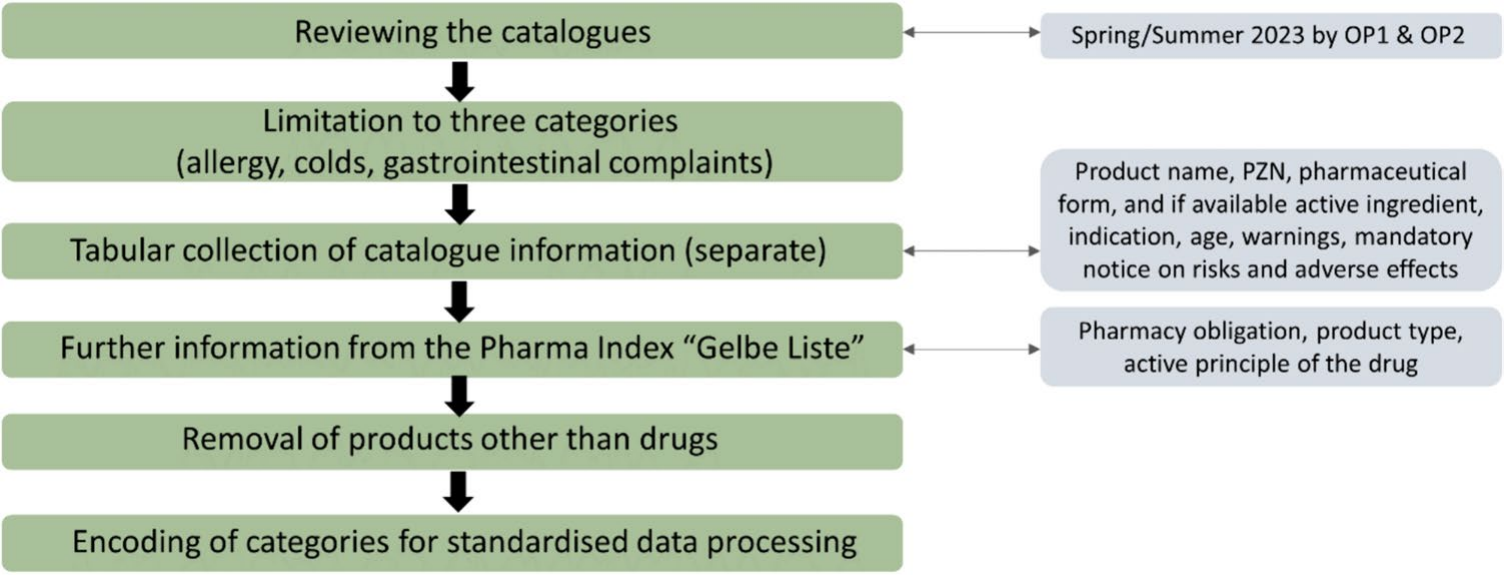
Graphical representation of the data collection process in a flow chart

For better data processing, the categories were encoded so that each subcategory of a category was redefined as a whole number; for example, the number 1 was assigned to the subcategory ‘tablet’ for the category ‘pharmaceutical form’. The categories ‘advertisement type’, ‘product type’, ‘mandatory notice’, ‘indications’, ‘pharmaceutical form’ and ‘active ingredient’ were encoded according to their subcategories. Figure 1 illustrates the data collection steps.

### Package leaflet

The law requires the content of the mandatory information to match the data in the package leaflet (Sect. 4 (2) of the HWG). For this reason, the information required by the mandatory information pursuant to Sect. 4 (1, 1a) of the HWG was checked for accordance with the information in the corresponding package leaflet. The package leaflets are available free of charge at https://www.gebrauchs.info/ and https://www.medikamente-per-klick.de/ (last accessed 24 January 2024).

### Synchronization and comparison

**Fig. 2:**
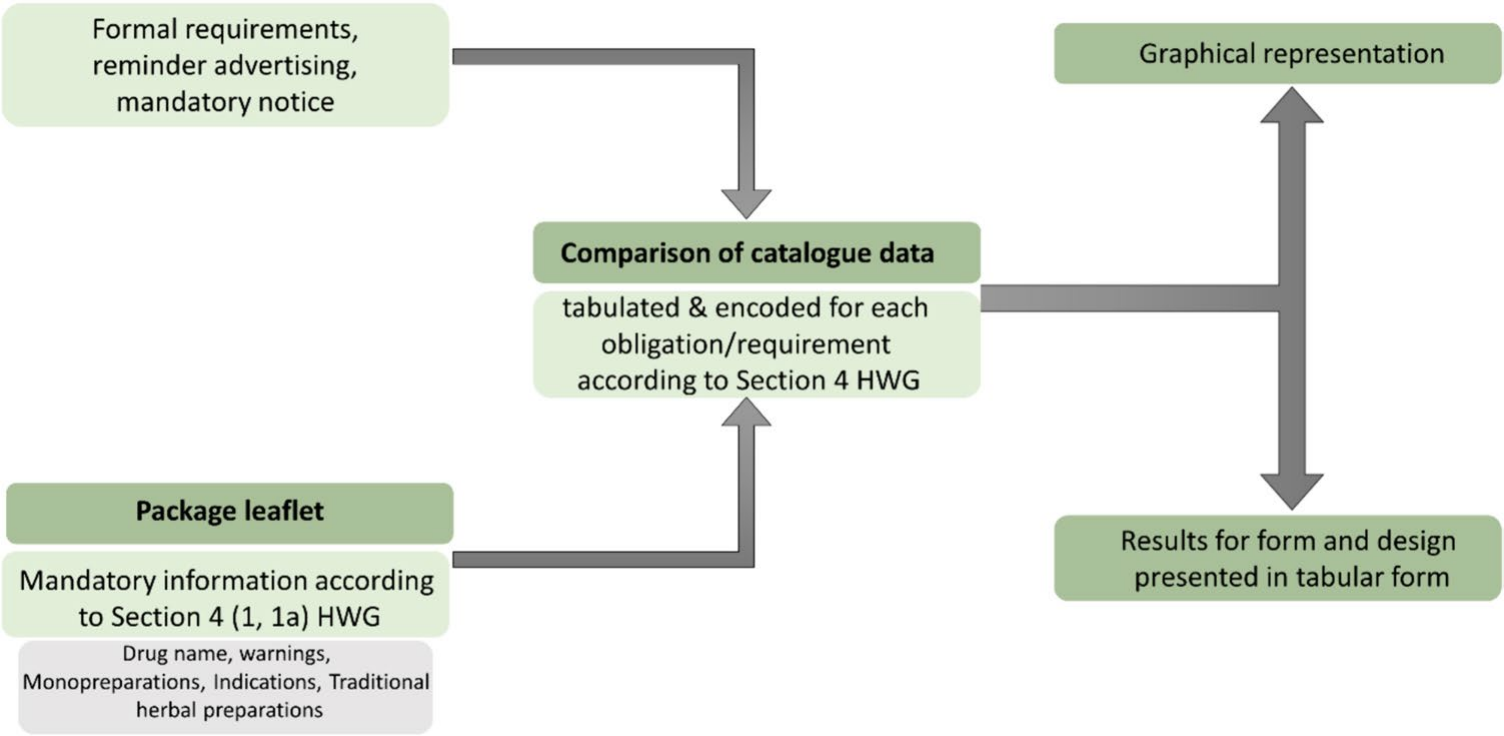
Graphical representation of the methods for comparing the catalogue data with the HWG in an arrow diagram

In the final step, the information from the catalogue was compared with the information from the package leaflet for the individual categories by encoding the results of the comparison and entering it in the data table. The codes are used for a uniform assessment of the extent to which the information in the catalogue and the package leaflet match. The meaning of the individual encodings is shown in the data table as a legend. The results for the availability of the mandatory notice on risks and adverse effects were added to the data table, whereas the results for the formal requirements and reminder advertising were recorded in separate tables. Finally, all results were presented graphically or in tabular form (Fig. 2).

## Results and discussion

### Comparison with the package leaflet

By focusing on the contents of the package leaflet for the mandatory information in advertising, we assessed whether reliable and objective information for the consumer as well as uniformity in advertising is provided (Bülow et al. 2012; Doepner and Reese 2023). This is why the mandatory information for the advertisement is based on the core information of the package leaflet, which can be legally influenced by the authorities in favour of objectivity (Bundesregierung der Bundesrepublik Deutschland 1975). In addition, consumers benefit from standardized information in both advertisements and package leaflet in the sense that they already know some of the information in the package leaflet when they purchase the drug based on the advertisement (Doepner and Reese 2023).

Moreover, the possibility of reproducing the mandatory information from the respective package leaflet in advertisements only analogously but not verbatim means that the wording can be better adapted to laypersons to make the advertisement easier to understand (Doepner and Reese 2023; Prütting 2016). However, this simplification harbours the risk that inappropriate wording can lead to inaccuracies and misunderstandings, which could be misleading and detrimental to drug safety (Doepner and Reese 2023).

### Brand name of the drug

**Fig. 3:**
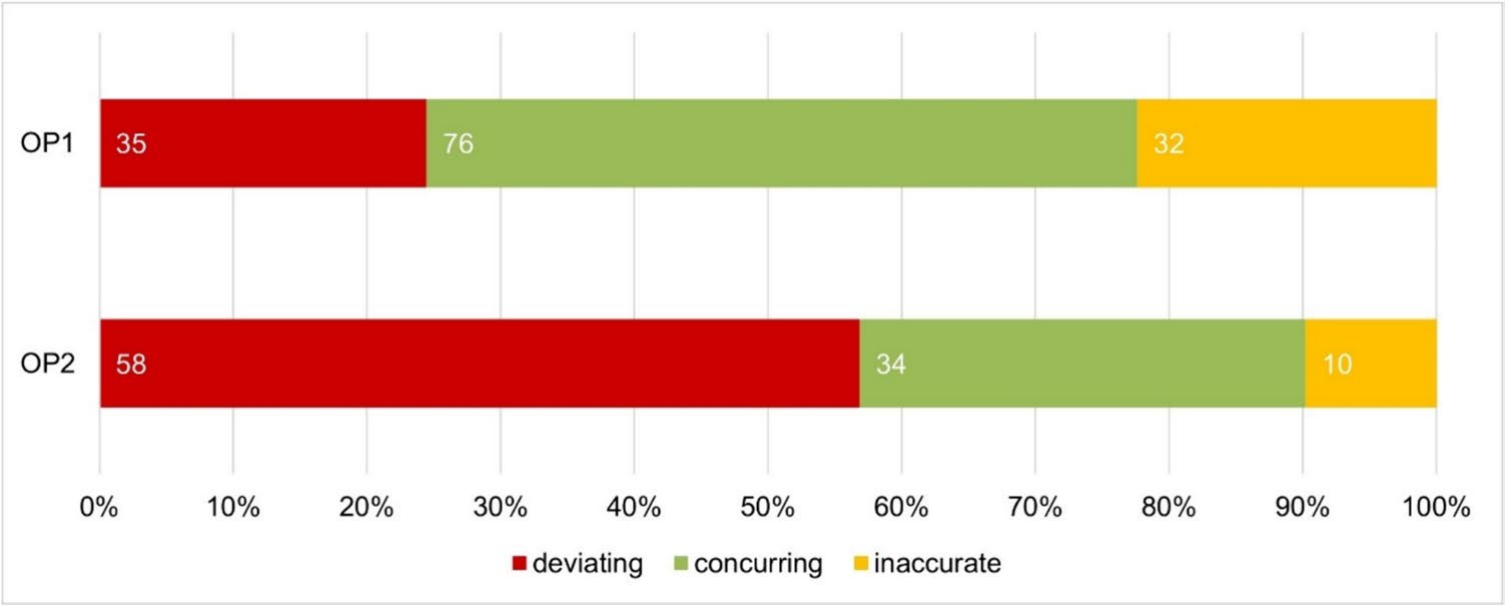
Comparison of the drug name given in the product catalogues of OP1 (top) and OP2 (bottom) with the brand name in the package leaflet in a bar chart; deviating information is shown in red; matching information is highlighted in green; inaccuracies of the brand names are marked in yellow

Figure 3 shows the comparison of the drug’s brand name between the provided information in the package leaflet and the information in the product catalogues of OP1 (top) and OP2 (bottom). In this paper, the name of the drug always refers to the brand name and not to the generic name. The brand name of the drug is essential for its identification and is already determined by the time of authorization (Sect. 22 (1) of the AMG). Therefore, the drug’s name in the advertisement must adhere completely to the brand name specified in the marketing authorization and must not deviate from it by abbreviation or otherwise (Doepner and Reese 2023; Prütting 2016). OP1 lists the brand name of the drug in 53.2% (76) of the products in accordance with the information on the package leaflet and OP2 in 33.3% (34).

The category of inaccuracies includes lowercase spelling of name components that should be capitalized, or the addition of pharmaceutical forms to the product name contrary to the brand name specified in the package leaflet. These inaccuracies of the brand names occur in 22.4% (32) of the products in OP1 and in 9.8% (10) of the products in OP2.

Serious deviations, such as the omission of brand name parts, affect a quarter of the product names in OP1 (24.5%; 35) and more than half (56.9%; 58) in OP2. Due to the deviation from the brand name of the drug specified in the package leaflet, there is a risk that the drugs cannot be clearly identified. Uncertainty and confusion can arise for consumers because the product they are looking for is known to them under a different name. There is also the risk of many different products falling under the same name. The deviation from the brand name of the drug is therefore an expression of consumer unfriendliness and an offence against the HWG.

Although a drug name was given to all products in both catalogues, according to the brand name specified in the package leaflet, 46.9% of them given in OP1 and 66.7% of them given in OP2 deviated from the specified brand name of the drug. Particularly in terms of consumer friendliness, this approach to stating the name of the drug is a violation of the mandatory information requirements of the HWG (Sect. 4 (1) sentence 1 no. 2 of the HWG).

### Indications

**Fig. 4:**
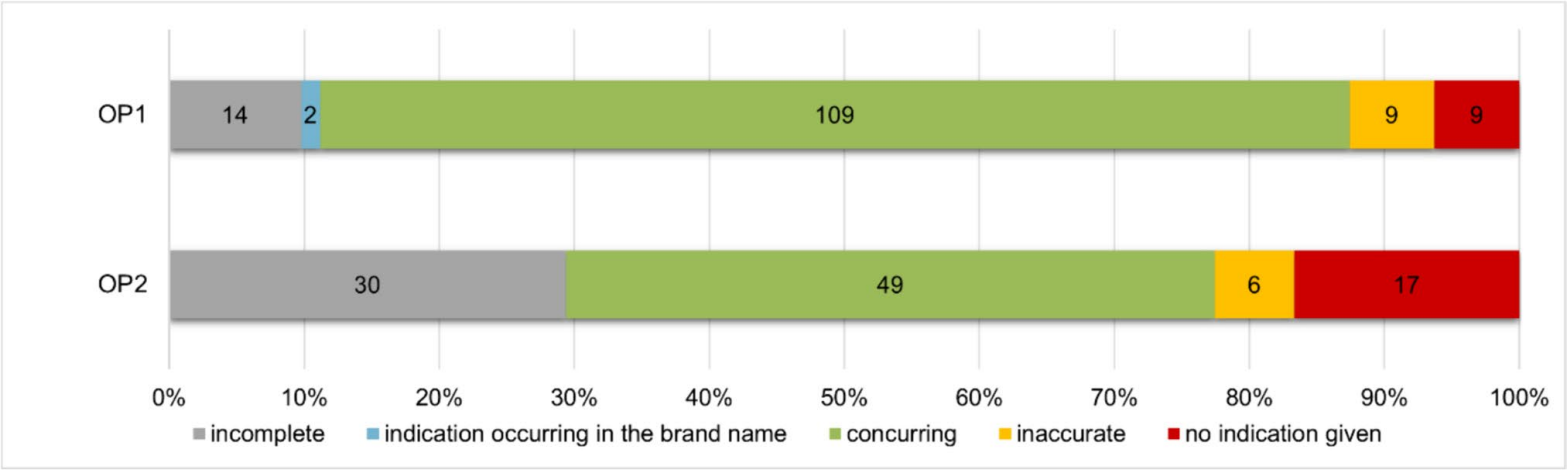
Comparison of the specification of the indications in the product catalogue of OP1 (top) and OP2 (bottom) in a bar chart. In grey, the absence of one or more indications; in blue, no indication, as already in the brand name; in green, matches; in yellow, inaccuracies; in red, the absence of any indications

Figure 4 shows the implementation of the indications for the respective drugs. The analysis is based on a comparison of the indications mentioned in the catalogue with the information given in the package leaflet. The indications for a drug are also specified by the time of authorization and reflect the possible application areas for this drug (Doepner and Reese 2023). Drug advertisements must only state the indications which are to be advertised or referred to, even though the drug can be used for several indications (Bülow et al. 2012). In addition, the two product catalogues are aimed at laypersons, which is why only indications that can be implemented in the form of self-medication should be listed in the product catalogue.

OP1 listed fewer indications for 9.8% of their advertised drugs compared to the number of listed indications in the package leaflet. OP2 did this for 29.4% of their advertised drugs. Moreover, the indication may be omitted if it is already part of the brand name (Bülow et al. 2012). This only occurs in 1.4% (2) of the OP1 advertisements.

There were inaccuracies in the indications when slight discrepancies in the descriptions of the indications between the product catalogue and the package leaflet occurred. For example, the catalogue stated the indication acute bronchitis, whereas the package leaflet stated the indication general respiratory diseases. However, as the law does not require the contents of the package leaflet and advertisement must match word for word, these inaccuracies can be tolerated (Doepner and Reese 2023). This affects 6.3% (9) of OP1 products and 5.9% (6) of OP2 products.

Nevertheless, the complete omission of specifying indications constitutes a violation of the mandatory information. As a result, the consumer is confronted with products in advertisements without being able to link them to a possible indication. This can lead to consumers buying drugs, which are inappropriate for their specific symptoms. In the best-case scenario, the consumer reads the package leaflet before taking the inappropriate drug for the first time and then refrains from taking it. In contrast, in the worst-case scenario, the consumer takes a drug that is not authorized for the desired application and will therefore be ineffective, as well as putting unnecessary strain on their body and metabolism. In addition, from an economic point of view, a misleading advertised indication leads to an unnecessary expense for the consumer and only creates a revenue for the distributor, as the consumer will possibly buy a more suitable drug in the next step and thus make another purchase. OP1 omitted the indication for 9 products (6.3%) and OP2 even for 17 products (16.7%). Consequently, they advertised in a consumer-unfriendly manner and violated the obligation to list the indications (Sect. 4 (1) sentence 1 no. 4 of the HWG).

OP1 provides matching information on the indications for 76.2% (109) of the products and OP2 for 48% (49). Overall, OP1 complied with the obligation to state indications 93.7% of the time and OP2 83.3% of the time (Sect. 4 (1) sentence 1 no. 4 of the HWG), as inaccuracies, incomplete indications and indications occurring in the brand name do not constitute a violation.

### Warning about ethanol

**Fig. 5:**
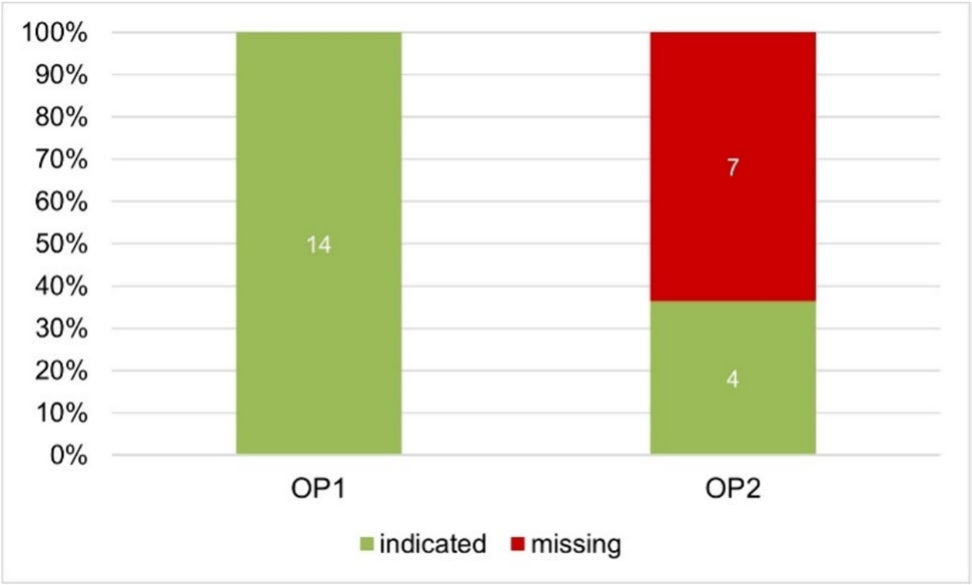
Comparison of the ethanol warning label in the product catalogue of OP1 (left) and OP2 (right) in a bar chart. In green, presence of the warning; in red, missing warning

Figure 5 shows the labelling of the ethanol warning in advertisements. The obligation to provide warnings serves to protect the consumer (Prütting 2016). The aim is to prevent negative consequences, for example by ingesting certain ingredients (Prütting 2016). This analysis only includes the ethanol warning. OP1 lists 14 products in its catalogue which have references to ethanol on the outer packaging. For 100% (14) of these drugs, OP1 lists the ethanol warning.

OP2 lists 11 products in its catalogue which have references to ethanol on the outer packaging and lists the warning for 36.4% (4) of these products in its catalogue.

Ethanol as an ingredient serves as a solvent, preservative or excipient in the drug, but harbours risks for certain patient groups, for example pregnant women, children, epileptics or patients with a history of liver or brain disease (Schuster 2017; https://www.apothekerkammer-nds-intern.de/index.php?did=26&view=4118,4&print=1; last accessed 31 January 2024). Consequently, it has to be openly communicated, if a product contains ethanol (Schuster 2017; https://www.apothekerkammer-nds-intern.de/index.php?did=26&view=4118,4&print=1; last accessed 31 January 2024). So, many patients need to avoid the ethanol ingredient. It is therefore unreasonable in terms of consumer protection to omit the warning in product catalogues like OP2 did in 63.7% (7) of their ethanol-containing products. There is a risk that these products will be purchased from the catalogue by consumers who think that the product does not contain ethanol since there is no warning in the advertisement. In the worst-case scenario, the consumer then takes the drug without reading the package leaflet, which can have various negative consequences depending on the patient group. Therefore, the violation of the obligation to include a warning by OP2 (Sect. 4 (1) sentence 1 no. 7 of the HWG) is serious.

### Traditionally registered drugs

In the case of herbal drugs which are not authorized but have been registered based on many years of usage, the statement ‘Traditionelles pflanzliches Arzneimittel zur Anwendung bei … (spezifizierte/-s Anwendungsgebiet/-e) ausschließlich auf Grund langjähriger Anwendung’ (Sect. 4 (1) sentence 2 of the HWG) must be listed. The statement is to be understood as mandatory (Grunert and Meyer, 2005; Prütting 2016).

**Fig. 6:**
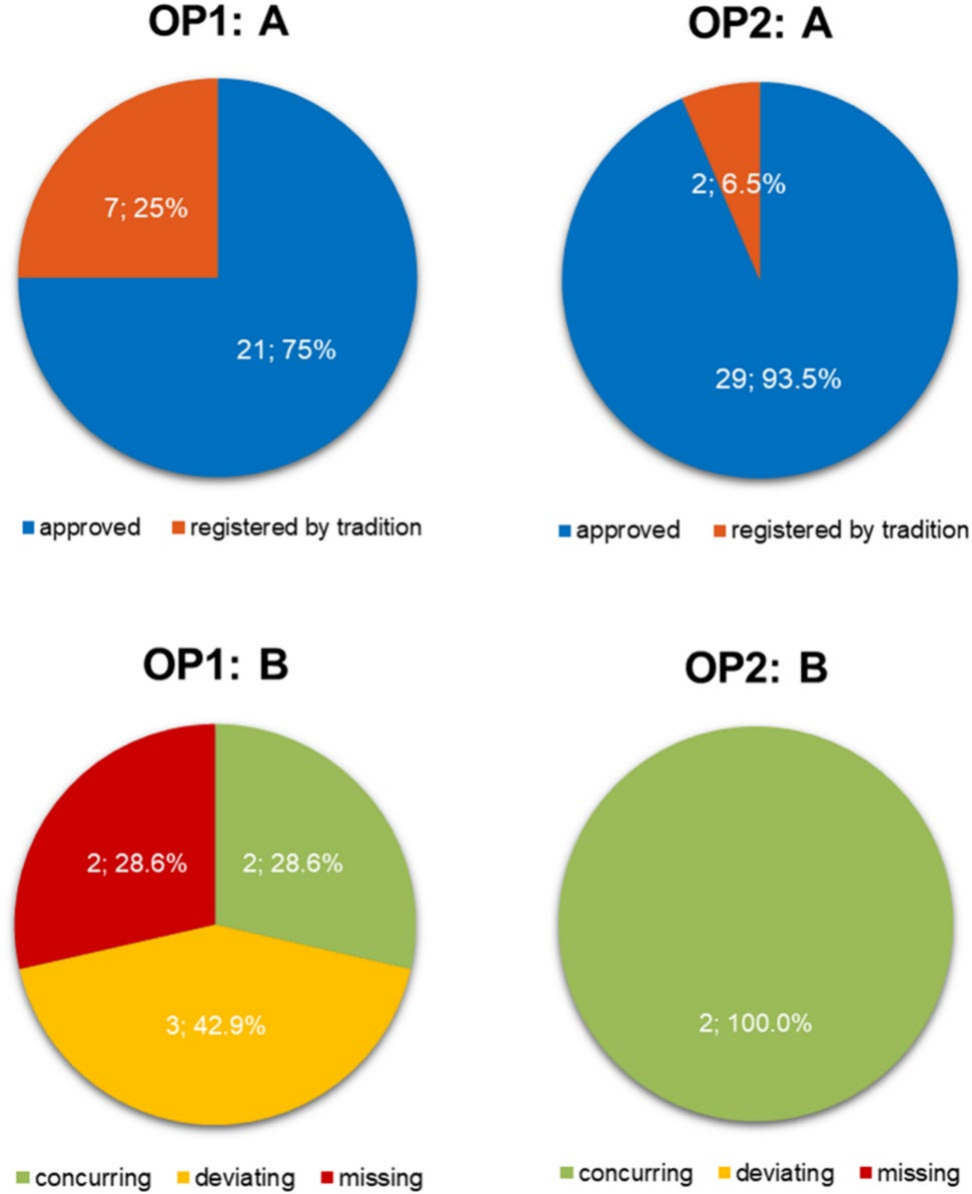
Left: OP1. Right: OP2. Part A: comparison of the classification of herbal preparations according to authorization or registration as traditional herbal drug in the product catalogue of the online pharmacies in two pie charts. In blue, authorized preparations; in orange, traditionally herbal registered preparations. Part B: type of specification of the mandatory reference to traditional herbal registered drugs for the respective traditional herbal drugs available in the product catalogue in two pie charts. In green, matching reference; in yellow, deviating reference; in red, missing reference

Drugs normally undergo a marketing authorization procedure if a manufacturer wishes to place them on the market to ensure that the drug meets certain standards in terms of safety and efficacy (Veit, 2014). If a drug proves its efficacy in the authorization procedure based on traditional lists and scientific bibliography for a specific indication, the drug can be registered as a traditional herbal drug (Reichling, 2008; Veit, 2014). As the application is based on tradition, it was stipulated that a drug or the active ingredient composition must have been used in a medical context for at least 30 years and, in addition, the application must have been known in the European Union for at least 15 years (Gemeinsamer Bundesausschuss, 2014). Furthermore, traditional herbal drugs may only be used for indications that do not require medical supervision (Bülow et al. 2012; Veit, 2014). Figure 6 shows in part A the classification of herbal preparations regarding authorization or registration as traditional herbal drug in the catalogue of OP1 (left) and OP2 (right). OP1 has 28 herbal-based products, of which 75% (21) were authorized and 25% (7) were registered based on tradition. OP2 advertises a total of 31 herbal drugs, of which 93.6% (29) received marketing authorization and 6.5% (2) were registered as traditional herbal drugs.

In the context of advertising, it appears even more important that the reference to traditional use (Sect. 4 (1) sentence 2 of the HWG) is mandatory. As to inform the consumer that the product is not based on clinical studies, long-term use justifies the marketing of the drug. Consequently, the labelling plays an important role in consumer friendliness and transparency.

Figure 6 part B illustrates the type of information on registered herbal drugs in the product catalogue of OP1 (left) and OP2 (right). The total quantity corresponds to the number of traditionally registered herbal drugs in the respective product catalogue.

OP1 omitted the reference in its product catalogue for 28.6% (2) of the traditionally registered products so that the consumer is not informed about the underlying registration. The consumer may mistakenly assume that the product has proven efficacy and therefore purchases it. In 42.7% (3) of the traditionally registered products, a statement was included, but this deviated from the legally stipulated wording in accordance with Sect. 4 (1) sentence 2 of the HWG. Deviations include the absence of the statement that the product is registered or the absence of the statement that the product was registered due to many years of use.

Just 28.6% (2) of the drugs included were correctly worded in OP1, whereas OP2 correctly formulated the information in 100% (2) of the concerned products.

### Monopreparations

The HWG does not require a complete list of the ingredients and/or active ingredients of the advertised preparations for laypersons. Only drugs with only one active ingredient, so-called monopreparations, must list the active ingredient in the advertisement (Sect. 4 (1a) of the HWG). A clear classification of the term ‘Wirkstoff’ must be stated before the active ingredient is named (Sect. 4 (1a) of the HWG).

**Fig. 7:**
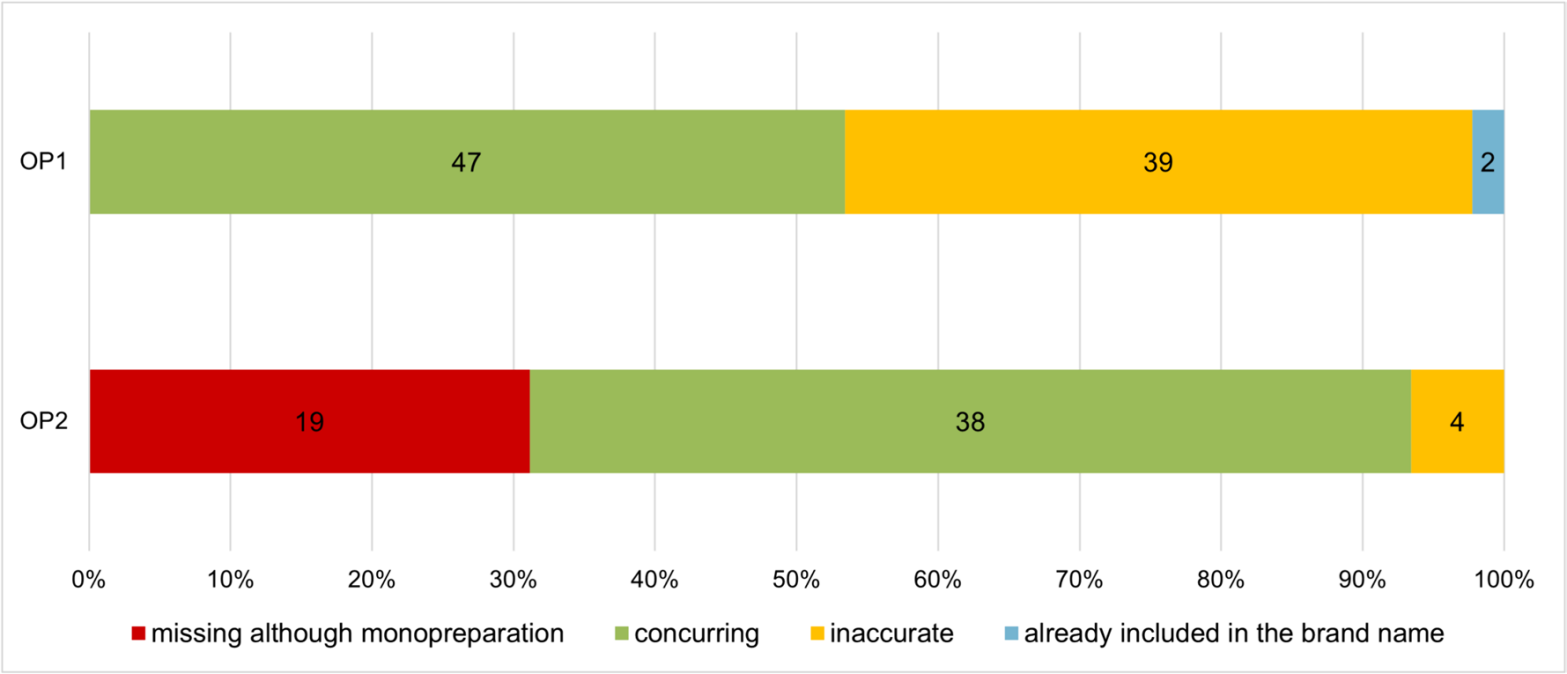
Comparison of the active ingredients stated for monopreparations in the product catalogues of OP1 (top) and OP2 (bottom) with the active ingredient information in the package leaflet in a bar chart. In red, missing information in the catalogue; in green, matching information; in yellow, inaccuracies in the information; in blue, no information, as contained in the brand name of the drug

Figure 7 shows the comparison of the active ingredient information for monopreparations between the information in the product catalogue of the two online pharmacies, OP1 (top) and OP2 (bottom), and the information in the package leaflet. More than half (53.4% (47)) of the monopreparations in OP1 and 62.3% (38) of them in OP2 are listed in the package leaflet. The law permits the omission of the active ingredient information if the active ingredient is listed as part of the brand name of the drug (Sect. 4 (1a) of the HWG). This is true for only 2 (2.3%) of the OP1 products.

Inaccuracies were found in 44.3% (39) of the active ingredient details of OP1 compared to 6.6% (4) inaccuracies in OP2. These included the specification of the active ingredient in metabolized form as a deviation from the approval data. For example, the specification ‘cetirizine’ was stated instead of ‘cetirizine dihydrochloride’. However, according to case law, analogous conformity is sufficient (Prütting 2016), so these inaccuracies in the active ingredient information may be tolerated and do not constitute an offence against the HWG.

Missing active ingredient information for monopreparations only occurs in OP2, where 31.2% (19) of monopreparations lack the information on the active ingredient. The specification of the active ingredient in monopreparations is intended to make the variety of drugs more transparent for the consumer (Die Bundesregierung der Bundesrepublik Deutschland 1986). Drugs containing the same active ingredient are sold under different brand names depending on the manufacturer, so that the brand name alone is not suitable for comparison, because it can be freely chosen by the manufacturer and is not predetermined (Bülow et al. 2012). Therefore, the specification of the active ingredient in monopreparations makes it easier for laypersons to compare the drugs on offer. The omissions in the OP2 catalogue are therefore a violation of the obligation to state the active ingredient for monopreparations (Sect. 4 (1a) of the HWG) and are also consumer-unfriendly. OP1 stated the active ingredient for all monopreparations and thus fulfilled this obligation.

### Mandatory notice and exceptions

The specification of the mandatory notice ‘Zu Risiken und Nebenwirkungen lesen Sie die Packungsbeilage und fragen Sie Ihren Arzt oder Apotheker’ (for risks and adverse effects ask your doctor or pharmacist) in advertisements for laypersons is based on the admission that some information, such as contraindications and adverse effects, may be omitted in advertising for laypersons (Sect. 4 (3) of the HWG). Therefore, the notice draws the consumer’s attention to the need to obtain information and not to automatically assume that an OTC drug cannot cause any risks or adverse effects simply because these were not stated in the advertisement.

Table 2 summarizes the legal requirements for the mandatory notice on risks and adverse effects in accordance with Sect. 4 (3) of the HWG. Italicized font means that the requirement has been implemented in a consumer-friendly and legally compliant manner. Bold font indicates implementations that are legally compliant but consumer-unfriendly.

**Table 2:** Explanation of the requirements for the mandatory notice on risks and adverse effects in accordance with Sect. 4 (3) of the HWG and comparison of the implementation of the requirements in the product catalogue of OP1 (third column) and OP2 (fourth column), shown in tabular form

Specification	Explanation	OP1	OP2
Performance of the mandatory notice	In the case of advertising for laypersons, the notice ‘Zu Risiken und Nebenwirkungen fragen Sie Ihren Arzt oder Apotheker’ must be included (Sect. 4 (3) sentence 4 of the HWG)	*- Notice completely available for all drugs by means of an asterisk*	*- Notice completely available for all drugs by means of an asterisk*
Localization of the mandatory notice	The mandatory notice must stand separately from other advertising contents (Sect. 4 (3) sentence 4 of the HWG)	*- The notice is located at the bottom right of each double page in the footer* *- The notice is distinct from other advertising contents* **- There is more information before and after the notice, so that it is not immediately obvious**	*- The notice is located at the bottom of each page in the footer (i.e. twice per double page)* *- The notice is separate from other advertising contents* *- The notice is the first item in the footer and is thicker than other information in the footer* *- The notice is followed by further information in the footer, which is not distracting, however, so that the notice is clearly visible*
Design of the mandatory notice	The mandatory notice must be clearly legible (Sect. 4 (3) sentence 4 of the HWG)	**- The notice is printed in a smaller font than the advertisement itself** *- Drugs are marked with two asterisks after the name and thus refer to the notice in the footer* *- Consumer-unfriendly, but still in accordance with the HWG*	*- The notice is printed in larger and thicker type than some parts of the advertisement itself and than other information in the footer* *- The headlines of the advertisements and/or the drugs are marked with an asterisk after the name and thus refer to the notice in the footer* *- Consumer-friendly and in accordance with the HWG*
Non-pharmacy-only drugs	In the case of non-pharmacy-only drugs, the information is only mandatory if the risks and adverse effects of these products are noted on the outer packaging or in the package leaflet (Sect. 4 (3) sentence 4 of the HWG)	*- The information was included in all non-pharmacy-only drugs, even if no risks or adverse effects were listed in the package leaflet*	*- The information was included for all non-pharmacy-only drugs, as risks or adverse effects were listed in the package leaflet for the products concerned*

Italicized font indicates consumer-friendly compliance with the requirements, and bold font indicates non-consumer-friendly compliance, but nevertheless compliant

OP1 and OP2 both use an asterisk to refer to the mandatory notice. The asterisk must be designed in such a way that the consumer can clearly recognize and track it (https://www.apothekerkammer-niedersachsen.de/berufsrecht_von_az.php?glossary=e#b3entry_aid1487; last accessed 4 February 2024).

The localization of the mandatory notice should be separate from other advertising contents (Sect. 4 (3) sentence 4 of the HWG). Both online pharmacies implement the demarcation by including the notice in the footer. OP2 includes the notice there in bold type as the first information on every page, so that the mandatory notice stands out there which can be seen as a consumer-friendly solution. In contrast, OP1 places the mandatory notice only once per double page and uses a smaller font size than in the advert itself. OP1 places further information and other notices before and after the mandatory notice, so that the mandatory notice does not immediately catch the consumer’s eye. This makes it more difficult for the consumer to follow the asterisk, and OP1’s method can therefore be categorized as consumer-unfriendly. Nevertheless, both procedures are legally compliant.

There is an exception to the mandatory notice, meaning that it is permitted to omit the mandatory notice for non-pharmacy-only drugs if no risks or adverse effects are listed in the package leaflet or on the outer packaging (Sect. 4 (3) sentence 4 of the HWG). Nevertheless, OP1 includes the mandatory notice for all drugs, even if no risks have been stated, while OP2 only advertises non-pharmacy-only products that have risks or adverse effects listed in the package leaflet, so that the mandatory notice is correctly included for all drugs.

In summary, the two online pharmacies (OP1 and OP2) complied with the statutory requirements for mandatory notice (Sect. 4 (3) of the HWG), although the latter chose a more consumer-friendly design and localization.

OP1 implemented Sect. 4 (3) of the HWG in accordance with the law, but in some respects, their solution is not consumer-friendly. In comparison, OP2 implemented Sect. 4 (3) of the HWG in a consumer-friendly manner.

### Formal requirements

The HWG provides formal principles to protect the reader (Sect. 4 (4) of the HWG) so that he should be able to find and perceive the medicinal-pharmacological characteristics of the drug directly (Doepner and Reese 2023). The purpose of these principles is to prevent the reader from being distracted by others, perhaps positively connoted advertising parts, which might lead to overlooking the informative and serious part (Prütting 2016). The formal requirements reflect the fact that the legislator wants to ensure the consumer friendliness of pharmaceutical advertising (Bülow et al. 2012).

Table 3 lists the formal requirements in accordance with Sect. 4 (4) of the HWG. Italicized font means that the formal requirement has been met.

**Table 3:** Explanation of the formal requirements in accordance with Sect. 4 (4) of the HWG and comparison of the implementation of the formal requirements in the product catalogue of OP1 (third column) and OP2 (fourth column), shown in tabular form

Formal specification	Explanation	OP1	OP2
Spatial demarcation	The advertising should be divided into 2 sections: an informative part and a part containing the other advertising claims (Doepner and Reese 2023)	*Separate mandatory information section follows directly under drug name; mandatory information in block form*	*Separate mandatory information section is listed at the bottom of each product advertisement; mandatory information in block form*
Possibility of design differentiation	Printing technique, border, font design, bullet points (Doepner and Reese 2023)	*Separator between drug name and mandatory information; font printed normally (not in bold or italics)*	*No border or separating lines; bullet points such as ‘Anw.’ and ‘Wirkstoff’ are printed in bold, and the following text is normal*
Font size	Evaluated in relation to other advertising components (Doepner and Reese 2023)	*Easy to read, same size*	*Easy to read, same size*
Mixing with other advertising contents	Mandatory information must not be mixed (Doepner and Reese 2023)	*Complied with*	*Complied with*
Horizontal arrangement	The mandatory information may not be placed vertically in print media (Doepner and Reese 2023)	*Complied with*	*Complied with*

Italicized font for compliance with the requirements

On the one hand, the law requires a clear distinction and demarcation with the aim of enabling consumers to identify the mandatory information as an informative, medical-pharmacological advertising part (Prütting 2016). It is about a strict spatial separation of the mandatory information in the sense of differentiation and the possibility of a creative differentiation from other existing advertising contents (Doepner and Reese 2023). Separating lines, borders, paragraphs and colour accents can be used for this purpose (Meier et al. 2018). The sections with the legally required mandatory information according to Sect. 4 of the HWG must not be mixed with other advertising sections in the slightest (Doepner and Reese 2023). Nevertheless, there is no obligation to issue an announcement prior to the individual mandatory information or to list the terms from the catalogue of mandatory information in accordance with Sect. 4 (1, 1a) of the HWG (Bülow et al. 2012). However, it is permitted to replace the terms from the mandatory information catalogue with similar, synonymous terms in bullet points (Bülow et al. 2012). Both online pharmacies separate the mandatory information in block form. OP1 uses a separating line between the name of the drug and the block of mandatory information, whereby the font is printed normally, which means it is not emphasized in bold or italics. OP2 uses neither a border nor a separating line, but bullet points such as ‘Anw.’ or ‘Wirkstoff’ are emphasized in bold in the mandatory information section. The mandatory information itself is not emphasized in bold or italics.

On the other hand, the law requires good legibility (Sect. 4 (4) of the HWG). This also includes the design, for example the choice of font colour and font size (Doepner and Reese 2023). The design should be adapted for an audience which includes people who do not have 100% visual acuity but can still read newspapers (Doepner and Reese 2023). It should also not require a lot of concentration to be able to read the information (Prütting 2016). The choice of font size for the mandatory information should not be assessed irrespective of the other advertising elements, but should always be considered in relation to them (Doepner and Reese 2023; Prütting 2016). In fact, the mandatory information should be recognized and read in the same way as other advertising elements (Doepner and Reese 2023). Both online pharmacies use the same font size for the mandatory information block as for other advertising texts. All mandatory information was listed horizontally, so that in both cases, good legibility and thus compliance with the specification can be assumed. In addition, neither OP1 nor OP2 listed any content in the mandatory information section other than the permitted information and therefore complied with the requirement. The product catalogue of both online pharmacies complied with and implemented the formal requirements of Sect. 4 (4) of the HWG.

### Reminder advertising

Strict legal requirements apply to reminder advertising, a shortened form of advertising. A reminder advertisement may not contain any medical-pharmacological content, such as indication or pharmaceutical form; otherwise, it is no longer a reminder advertisement (Doepner and Reese 2023). In addition to the brand name of the drug, the permissible information in a reminder advertisement includes the company, the name and the brand of the pharmaceutical company as well as the active ingredient (Sect. 4 (6) sentence 2 of the HWG; Doepner and Reese 2023). The specification of the active ingredient in a reminder advertisement is only permitted for monopreparations (Doepner and Reese 2023). In addition, information on package size and sales price is permitted as it does not contain any pharmacological content (Doepner and Reese 2023). However, as soon as the statements contain medical aspects, they are no longer reminder advertising (Doepner and Reese 2023). The categorization as reminder advertising is solely dependent on the information provided in the respective advertisement (Doepner and Reese 2023).

In the case of reminder advertising, the requirements of the HWG for advertisements regarding the mandatory information, the mandatory notice and monopreparations do not apply. Only information permitted by the law for reminder advertising is allowed (Sect. 4 (6) of the HWG). Therefore, listing indications as a part of the mandatory information is prohibited in reminder advertising (Doepner and Reese 2023). This also concerns indirect information about possible indications, for example in advertisement titles (Doepner and Reese 2023). The specification of the pharmaceutical form is also not permitted (Doepner and Reese 2023).

Table 4 lists 9 drugs from the OP1 catalogue and Table 5 lists 17 drugs from the OP2 catalogue which could potentially constitute reminder advertising in accordance with Sect. 4 (6) of the HWG. The tables show the information listed for each product in the advertisement in the catalogue. The selection of the analysed drugs is based on the absence of the indication in the advertisement in the catalogue, as its absence is an important criterion of reminder advertising. Taking all affected drug advertisements into consideration, both OP1 and OP2 could not be classified as reminder advertising in accordance with Sect. 4 (6) of the HWG. The reasons for this were either naming the pharmaceutical form, mentioning the mandatory notice on risks and adverse effects or the citation of titles that contained references to indications.

**Table 4:** Analysis of advertisements in the OP1 product catalogue that could fulfil the criteria for reminder advertising in accordance with Sect. 4 (6) of the HWG

Code of drug	Composition	Pharmaceutical form	Indication	Active ingredient	Pharmacy obligation	Mandatory notice
D01	Cromoglicic acid	**0**	**0**	**Cromoglicic acid, okay as an MP**	Yes	*Yes*
D02	Cromoglicic acid	**0**	**0**	**Cromoglicic acid, okay as an MP**	Yes	*Yes*
D03	Azelastine	**0**	**0**	**Azelastine, okay as an MP**	Yes	*Yes*
D04	Azelastine	**0**	**0**	**Azelastine, okay as an MP**	Yes	*Yes*
D05	Acetylsalicylic acid; pseudoephedrine	*Granules*	**0**	**0, not an MP**	Yes	*Yes*
D06	Acetylsalicylic acid; pseudoephedrine	*Granules*	**0**	**0, not an MP**	Yes	*Yes*
D07	Ascorbic acid; chlorphenamine; caffeine; paracetamol	*Granules*	**0**	**0, not an MP**	Yes	*Yes*
D08	Myrrh; chamomile flower dry extract; coffee charcoal	*Tablets*	**0**	**0, not an MP**	Yes	*Yes*
D09	Myrrh; chamomile flower dry extract; coffee charcoal	*Tablets*	**0**	**0, not an MP**	Yes	*Yes*

Exclusions for categorization as reminder advertising according to Sect. 4 (6) of the HWG are written in italics, and information written in bold is legally compliant; 0 means no specification

*MP* monopreparation

**Table 5:** Analysis of potential advertisements in the OP2 product catalogue that could fulfil the criteria for reminder advertising in accordance with Sect. 4 (6) of the HWG

Code of drug	Composition	Pharmaceutical form	References from title, if applicable	Indication	Active ingredient	Pharmacy obligation	Mandatory notice
D10	Dyer’s coneflower rhizome, purple coneflower root, pale coneflower root; arborvitae tips and leaves	*Tablets*	*Cold kit for vegans*	**0**	**0, not an MP**	Yes	*Yes*
D11	Paracetamol; phenylephrine	*Bag with powder*	*Cold kit for vegans*	**0**	**0, not an MP**	Yes	*Yes*
D12	Xylometazoline hydrochloride	*Nose spray*	*Cold kit for children*	**0**	**0**	Yes	*Yes*
D13	Pentoxyverine	*Drops*	*Cold kit for children*	**0**	**0**	Yes	*Yes*
D14	Levomenthol, racemic camphor	**0**	**0**	**0**	**0, not an MP**	no	*Yes*
D15	Dextromethorphan	**0**	**0**	**0**	**0**	Yes	*Yes*
D16	Levomenthol; racemic camphor; purified turpentine oil; eucalyptus oil	*Ointment*	**0**	**0**	**0, not an MP**	no	*Yes*
D17	Paracetamol/phenylpropanolamine/dextromethorphan	*Hard capsules*	**0**	**0**	**0, not an MP**	Yes	*Yes*
D18	Paracetamol; pseudoephedrine; pseudoephedrine; diphenhydramine	*Film-coated tablets*	**0**	**0**	**0, not an MP**	Yes	*Yes*
D19	Paracetamol/phenylpropanolamine/dextromethorphan	*Hard capsules*	**0**	**0**	**0, not an MP**	Yes	*Yes*
D20	Dextromethorphan, doxylamine, ephedrine, paracetamol	*Syrup*	**0**	**0**	**0, not an MP**	Yes	*Yes*
D15	Acetylcysteine	*Effervescent tablets*	*Cough set day & night*	**0**	**0**	Yes	*Yes*
D21	Dextromethorphan	*Syrup*	*Cough set day & night*	**0**	**0**	Yes	*Yes*
D22	Aconitinum D5, atropinum sulfuricum D5; hydrargyrum bicyanatum D8	*Drops*	**0**	**0**	**0, not an MP**	Yes	*Yes*
D23	Aconitinum D5, atropinum sulfuricum D5; hydrargyrum bicyanatum D8	*Globules*	**0**	**0**	**0, not an MP**	Yes	*Yes*
D24	Witch hazel leaves and twigs fresh distillate	*Ointment*	**0**	**0**	**0**	Yes	*Yes*
D25	Witch hazel leaves and twigs fresh distillate	*Suppository*	**0**	**0**	**0**	Yes	*Yes*

Exclusions for categorization as reminder advertising according to Sect. 4 (6) of the HWG are written in italics, and information written in bold is legally compliant; 0 means specification

Since reminder advertising is an abbreviated advertisement containing only a small amount of legally required information, there is no risk of misinforming the consumer and, therefore, no risk of misjudging the drug (Doepner and Reese 2023). However, if the scope of a reminder advertisement is extended beyond the permitted area, the entire catalogue of mandatory information (Sect. 4 (1) of the HWG) must be listed completely, as required by law. If that is not implemented, there is a risk that the consumer will not be adequately informed about the advertised drug due to the incomplete supply of mandatory information. As a result, there is a risk of misleading the consumer, which means that the protective function of Sect. 4 of the HWG can no longer be guaranteed.

Both OP1 and OP2 included further information from the mandatory information catalogue for the products examined in addition to the information permitted for reminder advertising. Hence, there is no reminder advertising. At the same time, however, they have not listed all the required information. Thus, the consumer might be misled and consequently misjudges the suitability of the respective drugs for his own purposes. In this regard, the HWG has not been complied with and OP1 and OP2 have committed an offence to the disadvantage of the consumer.

### Legal conformity of the catalogues

**Fig. 8:**
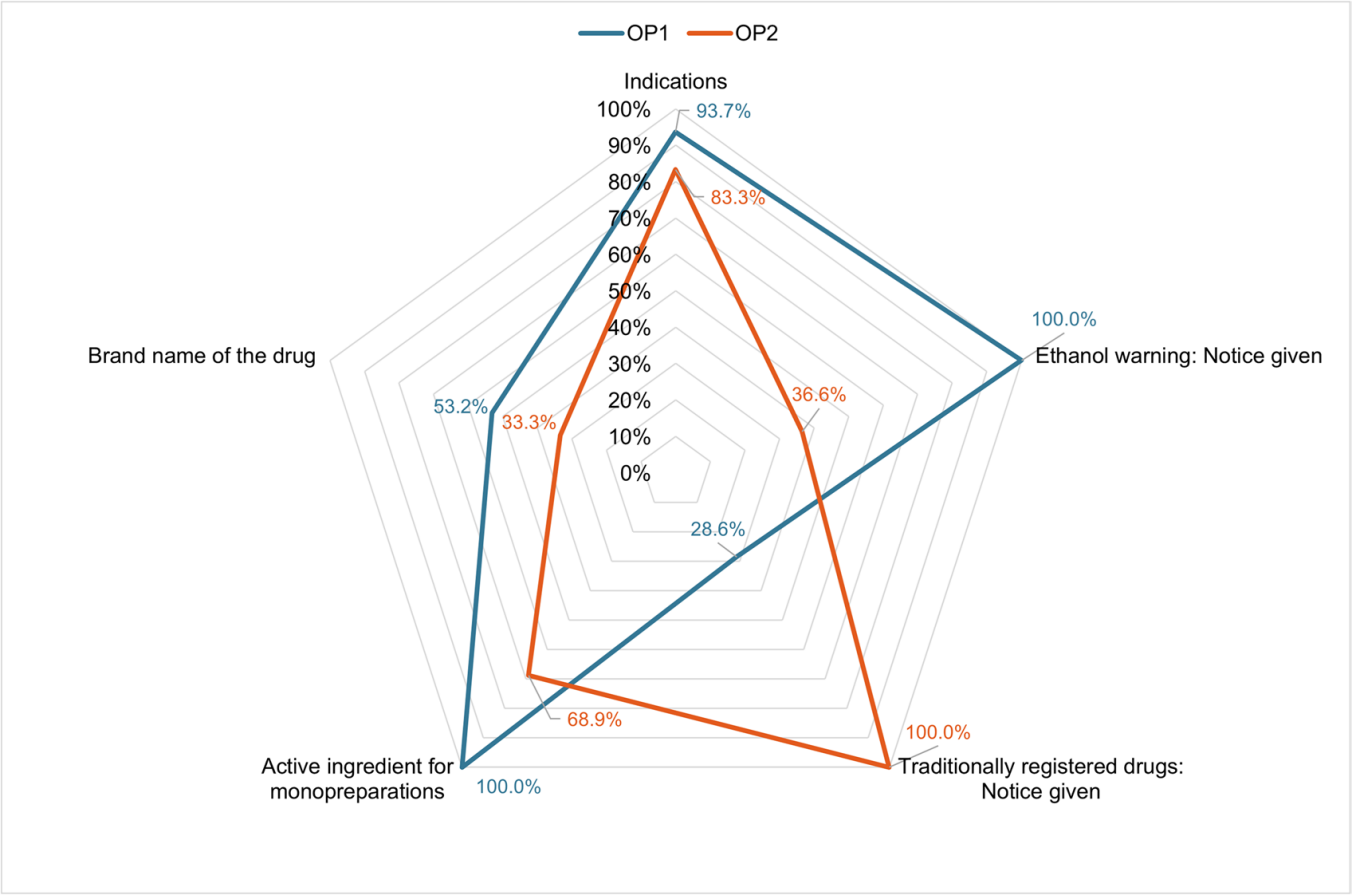
Graphic representation of the legally compliant listing of the mandatory information on indications, the ethanol warning, the reference for traditionally registered drugs, the active ingredient for monopreparations and the indication of the brand name of the drug in the product catalogues of OP1 (blue) and OP2 (orange) in a network diagram

In a direct comparison of the compliance with the previously presented mandatory information in accordance with Sect. 4 (1, 1a) of the HWG, the two catalogues differ greatly in terms of the proportion of correct execution of the individual disclosures. This is illustrated in Fig. 8. While the specification of the indications and the active ingredients for monopreparations are similarly well done, there are large differences in the specification of warnings and references to traditionally registered drugs. It is also noticeable that OP1 performs better than OP2 in four of the five categories (indications, ethanol warning, brand name of the drug, active ingredient). The average legally compliant information in the five categories is 75.1% for OP1 and 64.4% for OP2, whereby the inaccurate but still legally compliant information is included. Therefore, the HWG was only partially complied with and implemented, even though it is mandatory. There are legal deficiencies. At this point, the consumer-protecting function of Sect. 4 of the HWG should be emphasized again (Bülow et al. 2012). Its effectiveness cannot be fully guaranteed in case of inadequate compliance with and insufficient implementation of the HWG, which is true for the considered catalogues.

If a law exists but its implementation is not or only insufficiently monitored, the aim of the law is missed. In addition, there are legal uncertainties regarding the HWG, which could be exploited by advertisers for their own benefit in contrast to the intention of the HWG (https://der-arzneimittelbrief.com/artikel/1999/das-heilmittelwerbegesetz-und-der-verbraucherschutz; last accessed 26 March 2024). In 1998, such shortcomings in the implementation of the HWG were discussed at a theme day at the Bergische Universität, Gesamthochschule Wuppertal, under the direction of Prof. Dr G. Borchert (https://der-arzneimittelbrief.com/artikel/1999/das-heilmittelwerbegesetz-und-der-verbraucherschutz; last accessed 26 March 2024), which shows how long the problem has already existed and how profound it is. It is unclear why this problem has not been resolved since then and that the HWG can still be circumvented.

Section 7 of the German General Administrative Regulation on the Implementation of the Drug Act (*Allgemeine Verwaltungsvorschrift zur Durchführung des Arzneimittelgesetzes* (AMGVwV)) regulates the monitoring of the HWG. Section 7 (1) of the AMGVwV states that compliance with the HWG for drug advertisement should be ensured by the authority responsible for monitoring the AMG. These are the individual federal state authorities (https://www.deutsche-apotheker-zeitung.de/daz-az/2015/daz-8-2015/behoerdliche-ueberwachung; last accessed 13 May 2024).

In the future, the monitoring of compliance with the HWG should be better regulated and it should be considered whether a coordinating department exclusive for monitoring the implementation of the HWG should be established and extended at the Federal Institute for Drugs and Medical Products (*Bundesinstitut für Arzneimittel und Medizinprodukte* (BfArM)).

### Comparison of the World Health Organization ethical criteria with the HWG

In its ‘Ethical criteria for medicinal drug promotion’, the World Health Organization (WHO) has defined criteria that can be used to assess adequate and truthful pharmaceutical advertising (World Health Organization 1988). The criteria are to be understood as guidelines that can be adapted by the respective governments within national laws (World Health Organization, 1988). Therefore, the WHO criteria are not to be regarded as binding, but are rather intended to provide a framework for orientation (World Health Organization 1988).

Table 6 lists the WHO requirements from the ethical criteria for medicinal drug promotion in the first column. The second column lists the respective equivalent from the HWG. No requirements of the HWG that go beyond the WHO criteria are listed, as the aim of the table is to show how the WHO requirements have been integrated into the HWG. The HWG has strongly integrated the WHO criteria, and there exist only a few differences, which are to be accepted in the context of individualization and adaptation of the criteria to the needs of the country. For example, the explicit indication of risks and contraindications, as proposed by the WHO, has been omitted. Alternatively, however, the mandatory information on risks and adverse effects was established in order to eliminate the occasionally large number of adverse effects and contraindications, which could overwhelm the layperson, and instead to encourage consumers to inform themselves independently about the adverse effects and risks (Tebroke 2021). Nevertheless, it should be emphasized that, except for the manufacturer’s address, all the information proposed in the HWG for lay advertising has been integrated.

**Table 6:** Comparison of the WHO ethical criteria for medicinal drug promotion with the requirements of the HWG

WHO: ethical criteria for medicinal drug promotion (1988)	HWG	Conclusion
Objective: Laypersons should receive information in order to make a rational decision on the use of drugs (14)	Objective: The consumer should be empowered to make an informed purchasing decision by receiving sufficient information about the drug (Doepner and Reese 2023)	*Same requirement*
Prescription-only drugs should not be advertised (14)	Prescription-only drugs may not be advertised to laypersons (Sect. 10 (1))	*Same requirement*
Drugs for certain diseases that require qualified treatment should not be advertised (14)	Certain illnesses that must be treated by specialist personnel may not be advertised to laypersons (Sect. 12 (1) sentence 1 no. 1)	*Same requirement*
To prevent drug abuse, narcotics and psychotropic drugs should not be advertised (14)	Drugs based on psychotropic substances with abuse potential may not be advertised to laypersons (Sect. 10 (2))	*Same requirement*
Advertising should not target children (14)	Advertising for laypersons may not be directed at children under the age of 14 (Sect. 11 (1) sentence 1 no. 12)	*Same requirement*
Claims about the efficacy of drugs for a particular disease may only be made if substantiated (14)	Misleading information on effectiveness is prohibited (Sect. 3 (1) sentence 1 no. 1)	*Same requirement*
Restrictions on the use of a drug should be indicated (14)	No specification, but alternatively there is the mandatory notice	**HWG does not have this requirement**
In layperson’s language, only scientifically proven information should be reproduced, for example according to the approval data (15)	Meaningful reproduction of the content for a lay audience is permitted (Doepner and Reese 2023; Mand, 2016). The mandatory information in the advertising should correspond to the content of the package leaflet (Bülow et al. 2012; Doepner and Reese 2023)	*Same requirement*
No fear or pressure should be communicated in advertising (15)	Advertising statements must not imply any disadvantages from not taking a drug (Sect. 11 (1) sentence 1 no. 7)	*Same requirement*
Specification: names of the active substances, either as INN or as authorized generic name (16)	Mandatory information: statement of active ingredient only for monopreparations (Sect. 4 (1a))	***HWG has lower requirements***
Specification: brand name (16)	Mandatory information: brand name (Sect. 4 (1) sentence 1 no. 2)	*Same requirement*
Specification: main indications (16)	Mandatory information: indications that are to be advertised must be listed (Doepner and Reese 2023)	*Similar requirement*
Specification: main precautions and contraindications (16)	Mandatory information: no precautions or contraindications need to be stated for lay advertising, instead the mandatory notice on risks and adverse effects must be given (Sect. 4 (3))	***HWG has lower requirements***
Specification: main warnings (16)	Mandatory information: warnings listed on the outer packaging of the drug (Sect. 4 (1) sentence 1 no. 7)	*Similar requirement*
Specification: name and address of the manufacturer or distributor (16)	Not applicable for lay advertising (Sect. 4 (3))	**HWG does not have this requirement**
Price information should correspond to the truth (16)	No specification	**HWG does not have this requirement**

Requirements in italics are identical or similar, in bold-italics are deviating requirements and in bold are WHO requirements that are not represented in the HWG

In the future, it might make sense to refer to other international regulations in addition to national laws and to check drug advertising in Germany for conformity with these. For example, in addition to the WHO criteria, it would also make sense to refer to the ‘PAGB Consumer Code for Medicines’ of the Proprietary Association of Great Britain and thus compare international laws and guidelines.

### International context

This analysis documents that there are considerable weaknesses in the compliance with the HWG regarding drug advertisements. These shortcomings are reflected globally. Table 7 lists important papers on pharmaceutical advertising from various countries. These papers underline problems of inadequate compliance with and insufficient implementation of regulations as well as laws on pharmaceutical advertising. Furthermore, they show that the results of this study do not represent an isolated problem in Germany but a worldwide issue.

**Table 7:** Overview of important international papers on the topic of pharmaceutical advertising and comparison with our results

Authors	Journal	Country	Content	Numbers	Principal findings	Suggestions	Conflict of interest	Compared with our results
Sansgiry et al. (1999)	*Health Marketing Quarterly*	USA	14 different printed OTC advertisements from 3 consumer magazines were analysed by 5 clinical pharmacists using a questionnaire	14 printed advertisements	Existing laws are disregarded; sometimes misleading images; false promises; lack of information on risks and adverse effects; lack of scientific evidence; overall, more than 50% of advertisements lacked essential information about the product	Regulatory authorities must establish more regular controls on OTC advertising	Unknown	Concurring
Villanueva et al. (2003)	*The Lancet*	Spain	References cited as evidence for statements in advertisements on antihypertensives and lipid-lowering drugs were reviewed	38 advertisements, 125 statements in total	18% references could not be found; 36% of the references do not support the statement of the advertisement	Doctors should critically scrutinize advertisements	1 author is associated with various pharmaceutical companies	Concurring
Solhaug et al. (2006)	*Tidsskriftet (The Journal of the Norwegian Medical Association)*	Norway	50 selected pharmaceutical advertisements received by 3 general practitioners were analysed for the stated references and conformity with Norwegian pharmaceutical advertising regulations	50 printed advertisements	52% of claims in advertisements are flawed; 61% of references given from journal articles had conflicts of interest; only half of advertisements complied with Norwegian pharmaceutical advertising regulations; often embellishing information in advertisements; written pharmaceutical advertisements should not be used to inform physicians	None	None	Concurring
Rohra et al. (2006)	*Journal of Pharmaceutical Sciences*	Pakistan	Information from advertising brochures for drugs was critically compared by a physician/pharmacologist with medical knowledge from PubMed articles; in addition, 122 general practitioners were asked about their primary source of information on drugs	345 different advertisements for 182 medicines from various manufacturers	18% of the advertisements considered were classified as misleading; general practitioners prefer to obtain information about new drugs via pharmaceutical advertising	An institution is to be set up to monitor the ethical implementation of pharmaceutical advertising	Unknown	Concurring
Santiago et al. (2008)	*BMC Medical Informatics and Decision Making*	Switzerland	References to advertising claims were checked for accuracy, as this is required by Swiss regulations on therapeutic products; 2 reviewers checked 6 Swiss medical journals for drug advertisements for analgesic tablets, gastrointestinal drugs and psychotropic drugs	29 advertisements with at least 1 reference	53% of advertisements reviewed were not substantiated by the reference provided or the reference had a conflict of interest; national regulations are insufficiently complied with; doctors should not trust pharmaceutical advertisements	Legal measures such as fines should be established	None	Concurring
Gahalaut et al. (2014)	*Indian Journal of Dermatology, Venerology and Leprology*	India, USA	Advertisements for prescription drugs from an Indian medical journal and an American medical journal were compared for compliance with the WHO ethical criteria for medicinal drug promotion and the IFPMA Code of Pharmaceutical Marketing Practices	76 advertisements	WHO criteria were met by 25% of the American journal advertisements and 0% of the Indian journal advertisements; 77% of the American journal advertisements and 25% of the Indian journal advertisements complied with the IFPMA Code; physicians should critically scrutinize drug advertisements	It should be investigated whether doctors are aware that pharmaceutical advertising often fails to implement recognized guidelines properly	2 authors received fees for trials on behalf of pharmaceutical companies	Concurring
Prasad et al. (2019)	*Journal of Nepal Health Research Council*	Nepal	Pharmaceutical companies could participate voluntarily and were asked to send in 10 advertisements for their best-selling products. These were checked for conformity with the WHO ethical criteria for medicinal drug promotion	48 pharmaceutical companies and in total 372 drug promotional literatures	Most of the advertisements considered met 5 to 8 of 11 WHO criteria	Informing all persons involved in advertising about the WHO criteria, multiple screenings should be established, and an uncomplicated way to report false advertising should be developed; review of national laws	None	Concurring
Rode et al. (2022)	*Cureus*	Outpatient departments of a tertiary care hospital	Drug promotional literatures from various pharmaceutical companies were checked for conformity with WHO criteria	192 printed drug promotional literatures	Generic name, brand name, amount of active ingredient and manufacturer name were specified 100%; indications (91%); dosage schedule (60%); adverse effects (24%); precautions and warnings (36%); contraindications and interactions (20%); address of manufacturer (63%); reference to the scientific basis (53%); no one met all criteria	The responsible supervisory bodies in each country should ensure the enforcement of laws that contain adequate rules on the quality of drug promotional literatures	None	Concurring
Keuper and Seifert (2023)	*Naunyn–Schmiedeberg’s Archives of Pharmacology*	Germany	Analysis of 123 OTC advertisements from a German health magazine for conformity with the HWG	123 OTC advertisements	Lack of mandatory information in advertisements reflects non-compliance with the law	Control mechanisms for compliance with the law should be established	None	Concurring

*IFPMA* International Federation of Pharmaceutical Manufacturers and Associations

Consequently, solution strategies should not only be developed at a national level, but also internationally. The compliance with common guidelines and standards for pharmaceutical advertising, such as those issued by the WHO, should be more closely monitored and non-compliance more strictly prosecuted.

Many of the papers listed in Table 7 did not examine advertising for laypersons but advertising specifically for doctors. All these studies call for doctors not to be misled by alleged references to effectiveness, but to critically scrutinize advertising. The FDA has developed the so-called ‘Bad Ad Program’, which is an educational program for prescription drug advertising in the USA designed to train healthcare professionals to recognize and quickly report false or misleading prescription drug advertising (https://www.fda.gov/drugs/office-prescription-drug-promotion/bad-ad-program; last accessed 4 July 2024). What is progressive here is that the program is not only aimed at doctors, but also at all professional groups that could potentially meet pharmaceutical advertising (https://www.fda.gov/drugs/office-prescription-drug-promotion/bad-ad-program; last accessed 4 July 2024). An online workshop is offered in which, in addition to recognizing false advertising, the reporting of false advertising is also trained (https://www.fda.gov/drugs/office-prescription-drug-promotion/bad-ad-program; last accessed 4 July 2024). This program is a good example of the fact that advertising recipients are not powerless in the face of misleading advertising but can even help to prevent it.

As a first step, it would be advantageous for Germany for the BfArM to offer an uncomplicated way to report possible false advertising. Additionally, the BfArM should implement a similar program to educate German healthcare professionals and make them less susceptible to false advertising. It should be considered whether the topic of legally compliant pharmaceutical advertising, including the WHO criteria and national guidelines and laws, can be integrated into the curriculum for future recipients of pharmaceutical advertising, such as medical students, to raise awareness at an early stage. In the area of lay advertising, other options must be found to prevent the non-compliance of advertisements with legal regulations and international standards.

### Limitations of the analysis

The two authors have pharmacological and medical training but not formal legal training. The interpretation of the law was primarily based on Ulf Doepner and Ulrich Reese’s commentary on the Therapeutic Products Advertising Act from 2023. In addition, the interpretations of the Therapeutic Products Advertising Act by Bülow et al. (2012) and by Prütting (2016) were considered. As the commentary on the law is subjectively influenced by the authors, the views of these authors are reflected in the interpretation and in the assessment. Our results could be slightly altered by other interpretations, but the key message will remain the same.

The law does not require a literal match, but an analogous match. Since the assessment of what is ‘analogous’ is not entirely objective, a certain subjective component is included in the judgement. This also applies to the assessment of ‘imprecise’ and the assessment of the consumer-friendliness of the mandatory notice (Sect. 4 (3) of the HWG) as well as the formal requirements (Sect. 4 (4) of the HWG).

Our analysis of the warning’s compliance with Sect. 4 (1) sentence 1 no. 7 of the HWG was limited to warnings about ethanol.

The samples analysed in this paper represent only a portion of a larger product catalogue from the respective online pharmacy. But it can be reasonably assumed that the conclusions of this paper can be extrapolated to the remainder of the catalogues. Nevertheless, it should be noted that this study only covers a small sample size with one catalogue from each of two online pharmacies, which is why the results cannot be generalized.

Additionally, in case a drug was advertised in different package sizes, each package size was counted separately. Therefore, any deficiencies in the product were counted twice/multiple times, depending on how many package sizes exist for the respective drug.

## Conclusions

In both online pharmacy catalogues, Sect. 4 of the HWG was only insufficiently complied with concerning many parameters (Table 8). Our study shows that there are serious gaps in the proper execution of Sect. 4 of the HWG. Taking into account that the HWG has a protective function for the consumer (Bülow et al. 2012; Doepner and Reese 2023), the gaps in execution of and deviations from the HWG must be criticized. Incomplete information does not provide the consumer with sufficiently neutral and factual information about the medical-pharmacological characteristics of the drug, thereby impairing or even depriving the consumer of the opportunity to assess the drug’s suitability and, consequently, the necessity of purchasing it (Doepner and Reese 2023).

**Table 8:** Overview of the results of the analysis of the individual parameters

Analysed parameters	Concurring OP1 (%)	Concurring OP2 (%)	Imprecise OP1 (%)	Imprecise OP2 (%)	Deviating OP1 (%)	Deviating OP2 (%)
Brand name of the drug	*53.2*	*33.3*	**22.4**	**9.8**	***24.5***	***56.9***
Indications	*87.4*	*775*	6.3*	5.9*	***6.3***	***16.7***
Warnings	*100*	*36.6*	–	–	***0***	***63.6***
Traditionally registered drugs	*28.6*	*100*	**42.9**	**0**	***28.6***	***0***
Monopreparations	*55.9*	*62.3*	44.3*	6.6*	***0***	***31.2***
Mandatory notice	*Complied with*	*Complied with*				
Formal requirements	*Complied with*	*Complied with*				
Reminder advertising	Non-existent	Non-existent				

Values marked with an asterisk are legally compliant despite the imprecise implementation. Concurring information is presented in italics, imprecise information in bold and deviating information in bold-italics

As Keuper and Seifert noted in their study of ‘Apotheken-Umschau’ in 2023, compliance with the law on drug advertisements is not sufficiently monitored (Keuper and Seifert 2023), so that these gaps in the product catalogues of the two online pharmacies are not surprising. Stricter control procedures must be established in the future to penalize such violations of the HWG and thus act in the interests of the consumer. In addition, non-compliance with the HWG should have consequences for the advertiser in accordance with Sect. 15 (3) of the HWG to deter further non-compliance.

Further studies should analyse the websites, mail newsletters and social media posts of online pharmacies for compliance with the Therapeutic Products Advertising Act. It will also be very informative to analyse drug advertisements in television magazines and television, targeting predominantly senior people who are easy victim for misinformation and disinformation. In addition, pharmaceutical advertising in various media should also be checked for compliance with international standards, such as the WHO criteria.

Based on the results of this study and the recent study by Keuper and Seifert (2023), we assume that the scientific analysis of other types of drug advertisements will reveal multiple other serious cases of legal non-compliance with the Therapeutic Products Advertising Act. We hope that this type of research will improve the quality of drug advertisements and have doctors, pharmacists, patients and consumers look much more critically on these items.

## Data Availability

All source data for this work are available upon reasonable request.

## Abbreviations

AMG: Arzneimittelgesetz (Drug Act)
AMGVwV: Allgemeine Verwaltungsvorschrift zur Durchführung des Arzneimittelgesetzes (German General Administrative Regulation on the Implementation of the Drug Act)
BfArM: Bundesinstitut für Arzneimittel und Medizinprodukte (Federal Institute for Drugs and Medical Products)
FIP: International Pharmaceutical Federation
HWG: Heilmittelwerbegesetz (Therapeutic Products Advertising Act)
IFPMA: International Federation of Pharmaceutical Manufacturers and Associations
MP: Monopreparation
OP1: Online pharmacy 1
OP2: Online pharmacy 2
OTC: Over-the-counter
PZN: Pharmazentralnummer (pharmaceutical central number)
Sect.: Section
WHO: World Health Organization
